# A practical field extraction method for non-invasive monitoring of hormone activity in the black rhinoceros

**DOI:** 10.1093/conphys/cot037

**Published:** 2014-02-03

**Authors:** Katie L. Edwards, Hannah M. McArthur, Tim Liddicoat, Susan L. Walker

**Affiliations:** 1 North of England Zoological Society, Chester Zoo, Caughall Road, Upton-by-Chester CH2 1LH, UK; 2 Institute of Integrative Biology, University of Liverpool, Crown Street, Liverpool L69 7ZB, UK; 3 School of Life Sciences, University of Glasgow, University Avenue, Glasgow G12 8QQ, UK; 4 Thermo Fisher Scientific, Tudor Road, Manor Park, Runcorn WA7 1TA, UK

**Keywords:** Corticosterone, faeces, *in situ*, progesterone, solid-phase extraction, testosterone

## Abstract

Non-invasive hormone analysis is a vital tool in assessing an animal's adrenal and reproductive status, which can be beneficial to *in situ* and *ex situ* conservation. However, it can be difficult to employ these techniques when monitoring *in situ* populations away from controlled laboratory conditions, when electricity is not readily available. A practical method for processing faecal samples in the field, which enables samples to be extracted soon after defaecation and stored in field conditions for prolonged periods prior to hormone analysis, is therefore warranted. This study describes the development of an optimal field extraction method, which includes hand-shaking faecal material in 90% methanol, before loading this extract in a 40% solvent onto HyperSep™ C8 solid-phase extraction cartridges, stored at ambient temperatures. This method was successfully validated for measurement of adrenal and reproductive hormone metabolites in faeces of male and female black rhinoceros (*Diceros bicornis*) and was rigorously tested in controlled laboratory and simulated field conditions. All the hormones tested demonstrated between 83 and 94% and between 42 and 89% recovery of synthetic and endogenous hormone metabolites, respectively, with high precision of replication. Furthermore, results obtained following the developed optimal field extraction method were highly correlated with the control laboratory method. Cartridges can be stored at ambient (cool, dry or warm, humid) conditions for periods of up to 6 months without degradation, before re-extraction of hormone metabolites for analysis by enzyme immunoassay. The described method has great potential to be applied to monitor faecal reproductive and adrenal hormone metabolites in a wide variety of species and allows samples to be stored in the field for up to 6 months prior to analysis. This provides the opportunity to investigate hormone relationships within *in situ* populations, where equipment and facilities may previously have been limiting.

## Abstract (precis)

A practical field extraction method was developed and rigorously tested under controlled laboratory and simulated field conditions, to allow extracted faecal hormone metabolites to be stored on solid-phase extraction cartridges. Metabolites can be stored at ambient temperatures for 6 months before analysis for reproductive and adrenal hormones in the laboratory by enzyme immunoassay.

## Introduction

Endocrinology is a useful tool that is being widely used by researchers to gain an insight into the hormonal mechanisms underlying behaviour, reproduction and stress responses in a wide variety of species (Schwarzenberger, 2007; Ganswindt *et al.*, 2012). An insight into endocrine physiology can be used to investigate hormone–behaviour relationships (Whitten *et al.*, 1998; Soares *et al.*, 2010), reproductive processes (Schwarzenberger, 2007; Hodges *et al.*, 2010) and indices of stress (Mostl and Palme, 2002; Sheriff *et al.*, 2011) and can be used to understand how individuals respond to both natural and anthropomorphic challenges in their environment (Walker *et al.*, 2005; Bradshaw, 2007; Denver *et al.*, 2009). For endangered species, both in the wild and in captivity, these approaches can be hugely beneficial in facilitating a better understanding of species biology, optimizing welfare and aiding conservation (Cockrem, 2005; Wielebnowski and Watters, 2007; Cooke *et al.*, 2013).

Faecal measures of hormone concentration are becoming widely used, because they are relatively easy to obtain and can be collected non-invasively, thus minimizing the impact of sampling on the animal (Millspaugh and Washburn, 2004). Additionally, faecal samples have the added benefit that they can represent the hormonal state of the individual over the hours preceding sample collection (Palme *et al.*, 2005), and can be practical for longitudinal sampling of individuals (Touma and Palme, 2005). However, faecal material has been processed by the body to aid excretion, so the resulting metabolites present in faeces may not be in their native form, as would be circulating the body (Palme, 2005; Touma and Palme, 2005). Excreted metabolites are vulnerable to bacterial and environmental degradation, which can further alter the metabolites present (Wasser *et al.*, 1988; Beehner and Whitten, 2004); but as long as faecal samples are collected relatively soon after defaecation, the hormone metabolites present can be a reliable indicator of the individual's physiological state (Palme *et al.*, 2005; Touma and Palme, 2005).

Often, the best option for sample preservation is to freeze samples immediately following excretion, which halts degradation and preserves hormone metabolites for long periods of time (Whitten *et al.*, 1998; Palme, 2005; Ziegler and Wittwer, 2005). However, field researchers may find themselves without reliable electricity supplies; therefore, alternative methods of sample preservation must be sought. Several methods have been used, including storage of faecal material in alcohol (Wasser *et al.*, 1988; Lynch *et al.*, 2003; Galama *et al.*, 2004), the drying of either raw faecal material (Brockman and Whitten, 1996; Galama *et al.*, 2004; Gould *et al.*, 2005) or faecal extract in the field (Santymire and Armstrong, 2010) and storage of faecal extract on filter paper (Shideler *et al.*, 1995); however, each method has potential constraints.

For example, storage of faeces in alcohol may prevent further degradation of hormone metabolites (however, see Khan *et al.*, 2002; Hunt and Wasser, 2003), but containers are vulnerable to leaking, and transportation of samples contained in a solvent from the field back to the laboratory can be problematic (Ziegler and Wittwer, 2005). The drying of samples prior to storage removes these two issues, but often still involves equipment to dry the faecal material, which may also require electricity. Furthermore, it can take hours or days to achieve dry samples, depending on field conditions, which leaves them vulnerable to contamination, sample loss and degradation during this time. Alternatively, it may be possible to analyse hormone metabolites by enzyme immunoassay (EIA) in the field (MacDonald *et al.*, 2008; Freeman *et al.*, 2010), but results are often qualitative rather than quantitative, and current methods have so far been limited to reproductive cyclicity and pregnancy diagnosis.

A solution may be the use of solid-phase extraction (SPE) cartridges; these are small, polypropylene cartridges containing a sorbent material, such as carbon–silica gel. These cartridges are often used for sample pre-treatment, to separate a desired analyte from liquid media, such as water, blood and urine, and are a common preparation step for high-performance liquid chromatography. The analytes of interest within a sample are separated from impurities based on their chemical properties. Analytes are selectively adsorbed and retained on the solid phase, allowing impurities to be washed away before re-extraction with a suitable solvent. Solid-phase extraction cartridges have previously been used for the field extraction of faecal samples (Stavisky *et al.*, 1995; Beehner and Whitten, 2004; Pappano *et al.*, 2010; Santymire and Armstrong, 2010), and appear to be an emerging tool for faecal extractions in the field. However, some studies have found unacceptable recovery of steroid hormones when storing extracts on these cartridges (Santymire and Armstrong, 2010), whereas others have found relatively good recovery (Ziegler and Wittwer, 2005).

In the present study, there was a desire to develop a field extraction technique for researchers who may have to track individuals over long periods of time, potentially needing to travel long distances, in environments where electricity supplies may be unreliable or non-existent. Freezing faecal samples upon collection is not always feasible, and existing field methodologies are not versatile enough to accommodate all of these requirements. Additionally, while existing methodologies often validated techniques for a single hormone, there was a desire to look at multiple hormones in the black rhinoceros (*Diceros bicornis*), to understand the reproductive state of individuals *in situ* and to investigate adrenal activity between individuals and populations, in order to maximize population performance.

Further development was therefore warranted, and the aim of this study was to develop a field extraction method that required minimal equipment and could be used where no electricity was available. The technique was designed to meet the following requirements: (i) to provide reasonable recovery of all synthetic and faecal hormone metabolites of interest; (ii) to provide accurate and repeatable quantitative results for faecal reproductive and adrenal hormone metabolites; (iii) to be stored at ambient temperatures in the field, without the risk of faecal hormone metabolite degradation before further processing in the laboratory; and (iv) to deliver results qualitatively comparable to controlled laboratory protocols.

## Methods

### Faecal samples and extraction

Faecal samples were collected from two male (age, 11 and 12 years) and three female (age, 8, 12 and 22 years) captive black rhinoceros (*D. b. michaeli*), housed at Chester Zoo, UK. Samples were collected immediately after defaecation, thoroughly mixed, separated into multiple bags to prevent repeated freeze–thawing and frozen at −20°C until required. All faecal samples were extracted in parallel using a control laboratory method (Walker *et al.*, 2002) and the newly developed field extraction method. In brief, for the control laboratory method, each sample was thawed, thoroughly mixed and weighed (0.5g ± 0.003 g), before adding 5 ml of 90% methanol, vortexing and shaking overnight on an orbital shaker. Each sample was then vortexed and centrifuged for 20 min at 598***g***. The supernatant was decanted, dried under air, resuspended in 1 ml of 100% methanol and stored at −20°C until analysis.

For the field extraction method, each sample was thawed, thoroughly mixed, and 0.5 g (±0.05 g) was weighed using portable battery-operated scales, suspended in 4 ml 90% methanol, and individually hand-shaken for 5 min. To separate the extract from the faecal material, samples were filtered using moistened filter paper (Grade 4, Whatman International Ltd., Maidstone, UK). The resultant faecal extract was then ready to load onto SPE cartridges, according to the optimal conditions outlined below.

In order to ensure that this modified faecal extraction method did not impact the recovery of hormone metabolites, a single faecal sample from one male and one female black rhinoceros were each thoroughly mixed, divided into five sub-samples and extracted according to the following methods. Faecal samples were treated as follows: (i) hand-shaken in 4 ml of 90% methanol for 5 min (*n* = 2 male and *n* = 2 female); (ii) hand-shaken for 5 min in 4 ml of 90% methanol, left to sit for 1 h, then hand-shaken for a further 5 min (*n* = 2 male and *n* = 2 female); or (iii) shaken overnight on an orbital shaker in 5 ml 90% methanol (*n* = 1 male and *n* = 1 female). The extract was then separated from the faecal material either by filtration [methods (i) and (ii)] or by centrifugation [method (iii)], evaporated to dryness, resuspended in 1 ml of 100% methanol and assayed in duplicate on the respective EIAs.

### SPE cartridges and optimal loading solvent

Two types of SPE cartridge were used for this study; both containing porous silica with a bonded alkyl chain, for hydrophobic retention [HyperSep™ octyl bonded silica (C8) and HyperSep™ octadecyl bonded silica (C18); 500 mg/3 ml; Thermo Fisher Scientific, Runcorn, UK]. Cartridges were primed according to the manufacturer's instructions (Thermo Scientific, 2011) with 4 ml of methanol followed by 4 ml of distilled water, with an average flow rate of 1 ml/min. Once primed, cartridges were loaded (0.5 ml/min) with either synthetic hormones or filtered black rhino faecal extract, using the optimal loading solvent as determined below. Cartridges were then washed with 2 ml of distilled water (1 ml/min), and sealed with Parafilm^®^ to prevent the solid phase from drying out during storage. Once ready for elution off the cartridge, 5 ml of 100% methanol was pushed through the column (0.5 ml/min), collected, dried under air, resuspended in 1 ml of 100% methanol and stored at −20°C until analysis.

The optimal solvent concentration for loading the extract onto the cartridge was determined by loading (0.5 ml/min) the synthetic hormones progesterone (P0130; Sigma Aldrich, UK; 200 ng), testosterone (T1500; Sigma Aldrich, UK; 300 ng) and corticosterone (C2505; Sigma Aldrich, UK; 500 ng) in 4 ml of distilled H_2_O onto primed C8 and C18 cartridges. The hormones were then eluted using a 10% stepwise increase in methanol concentration (5 ml; from 10 to 100% methanol). Each resulting 5 ml fraction was collected separately, dried under air, resuspended in 1 ml of 100% methanol and an aliquot quantified on the respective EIA. The percentage recovery of each synthetic hormone was calculated from the total concentration observed (in picograms per well) across all fractions eluted, as a percentage of the total mass of hormone expected (in picograms per well).

### Faecal extract recovery and precision

To determine the relative recovery and consistency of the field extraction method using endogenous material, a single faecal sample from one male and one female black rhinoceros were each thoroughly mixed before being divided into sub-samples. Sub-samples were then extracted according to either the control laboratory method (*n* = 10 male sub-samples; *n* = 10 female sub-samples) or the field extraction method and loaded onto primed C8 (*n* = 10 male sub-samples; *n* = 10 female sub-samples) or C18 cartridges (*n* = 10 male sub-samples; *n* = 10 female sub-samples), using the optimal loading solvent as outlined in the previous subsection. All resulting extracts were run on the respective EIAs. The percentage recovery of each faecal hormone metabolite was calculated from the observed concentration (in picograms per well) from each sub-sample stored on C8 or C18 SPE cartridges, as a percentage of the observed concentration (in picograms per well) from each sub-sample extracted following the control laboratory method. The recovery of the two cartridge types was compared using an independent samples *t*-test, in IBM^®^ SPSS^®^ Statistics version 20.

### Storage of hormone metabolites

A single faecal sample from one male and one female black rhinoceros were each divided into sub-samples, extracted according to the optimal field extraction method and loaded onto primed C8 cartridges (*n* = 25 male sub-samples; *n* = 25 female sub-samples). The cartridges were eluted on day 0 (*n* = 5 male; *n* = 5 female) or placed upright in cardboard storage boxes and then into airtight containers and stored in either cool, dry (range 15–25°C and 46–72% humidity inside container; *n* = 20 male; *n* = 20 female) or in warm, humid ambient conditions (range 19–36°C and 42–83% humidity inside container; *n* = 20 male; *n* = 20 female). The temperature and humidity inside the storage box were recorded every hour with a data logger. The cartridges were stored in this way for up to 6 months.

Hormone metabolites were eluted from cartridges at intervals (days 0, 30, 60, 90 and 180; *n* = 5 for each condition) and analysed using the respective EIAs. For comparison, the same male and female faecal samples were also treated as follows: (i) extracted at day 0 following the control laboratory method and extracts stored at −20°C; and (ii) faecal sub-samples stored at −20°C were also re-extracted (*n* = 5) following the control laboratory method at each storage interval.

### Comparison of laboratory and field protocols

Faecal samples collected approximately every other day for 6 weeks from one male (*n* = 20) and one female black rhinoceros (*n* = 20) were each mixed thoroughly, separated into two sub-samples, extracted in parallel according to the control laboraotry method or the optimal field extraction method and loaded onto primed C8 cartridges. Both sets of samples were analysed on the respective EIAs to allow quantitative and qualitative comparison of the two methods. To determine the accuracy of data following the field extraction method compared with the control laboratory method, a regression between the two sets of data was conducted in IBM^®^ SPSS^®^ Statistics version 20.

### Enzyme immunoassay

Synthetic hormones and faecal progesterone, corticosterone and testosterone metabolites were analysed using previously described enzyme immunoassays (Young *et al.*, 2004; adapted from Munro and Stabenfeldt, 1984), with some modifications. Each EIA used an antiserum (monoclonal progesterone CL425, polyclonal corticosterone CJM006 or polyclonal testosterone R156/7); corresponding horseradish peroxidase-conjugated label (C. J. Munro, University of California, Davis) and standards (Sigma-Aldrich, UK) on a Nunc-Immuno Maxisorp (Thermo-Fisher Scientific, UK) microtitre plate.

For progesterone and corticosterone, the procedure was as follows: (i) 50 μl per well of antiserum (1:10 000 for progesterone or 1:15 000 for corticosterone diluted in coating buffer) was loaded and incubated overnight at 4°C; (ii) plates were washed with wash solution (0.15 m NaCl, 0.05% Tween 20) five times; and (iii) standards (progesterone, 0.78–200 pg per well or corticosterone, 3.9–1000 pg per well) or samples diluted in EIA buffer were loaded at 50 μl per well; followed by (iv) 50 μl per well of horseradish peroxidase (1:35 000 for progesterone or 1:70 000 for corticosterone diluted in EIA buffer).

For testosterone, the procedure was as follows: (i) non-specific goat anti-rabbit γ-globulin (IgG; R2004; Sigma) was diluted in coating buffer, then loaded, 1.0 μg in 250 μl per well, on microtitre plates and incubated overnight at room temperature (RT). The non-specific IgG was then discarded, and 300 μl per well of Tris blocking buffer (0.02 m Trizma, 0.300 m NaCl and 1.0% bovine serum albumin, pH 7.5) was added and incubated for a minimum of 2 h at RT; (ii) plates were washed with wash solution five times; (iii) EIA buffer was loaded at 50 μl per well; (iv) standards (2.3–600 pg per well) or samples diluted in EIA buffer were loaded at 50 μl per well; followed by (v) 50 μl per well of horseradish peroxidase (1:40 000 in EIA buffer); and (vi) 50 μl per well of antiserum diluted 1:25 000 in EIA buffer.

Following incubation in constant light (progesterone) or in the dark (corticosterone and testosterone) for 2 h at RT, plates were washed with wash solution five times and incubated with 100 μl per well of RT substrate [0.4 mm 2,2′-azino-di-(3-ethylbenzthiazoline sulfonic acid) diammonium salt, 1.6 mm H_2_O_2_ and 0.05 m citrate, pH 4.0) and left to develop at RT in constant light (progesterone) or in the dark (corticosterone and testosterone). Following incubation, developed plates were measured at 405 nm.

Enzyme immunoassays were biochemically validated for measuring progesterone (1) and glucocorticoid metabolites (2) in female black rhino faecal extract, as well as testosterone (3) and glucocorticoid metabolites (4) in male black rhino faecal extract, through parallelism [(1) *R*^2^ = 0.970, *F*_1,6_ = 192.439; (2) *R*^2^ = 0.977, *F*_1,5_ = 212.34; (3) *R*^2^ = 0.989, *F*_1,6_ = 518.911; and (4) *R*^2^ = 0.969, *F*_1,5_ = 153.833; all *P* < 0.001] and matrix interference assessment [(1) *R*^2^ = 0.998, *F*_1,8_ = 3353.931; (2) *R*^2^ = 0.998, *F*_1,8_ = 4231.888; (3) *R*^2^ = 0.997, *F*_1,8_ = 2366.398; and (4) *R*^2^ = 0.995, *F*_1,8_ = 1498.983; all *P* < 0.001]. The cross-reactivities for progesterone, testosterone and corticosterone antibodies have been reported elsewhere [Walker *et al.*, (2008), de Catanzaro *et al.* (2003) and Watson *et al.* (2013) respectively]. Intra- and inter-assay coefficients of variation (CVs) were 7.32 and 8.47% for progesterone, 10.43 and 10.38% for testosterone and 6.92 and 11.69% for corticosterone EIAs, respectively. Synthetic standards and male or female black rhino faecal extracts eluted from cartridges were diluted as necessary in EIA buffer and run in duplicate (50 μl) on respective EIAs.

## Results

### Faecal extraction

Following the three faecal extraction methods, there were no differences in hormone metabolites measured in male or female faecal extracts (Median test: progesterone, *P* = 0.082; female corticosterone, *P* = 0.233; testosterone, *P* = 0.233; and male corticosterone, *P* = 0.233). This indicates that the field extraction protocol of hand-shaking for 5 min in 4 ml of 90% methanol is comparable to the control laboratory protocol, and given that this was deemed the most practical option for conducting in the field, was used for the remainder of method development.

### Synthetic recovery and optimal loading solvent

For both C18 and C8 cartridges, corticosterone eluted at between 50 and 60% methanol, followed by testosterone at 70% and progesterone at 80%. Therefore, to maximize the binding of hormone metabolites to the solid phase and minimize the risk of metabolites simultaneously being eluted in the loading step, the optimal loading solvent concentration for all subsequent experiments was adjusted from 90% methanol down to 40% methanol.

The recovery of synthetic progesterone was similar for the C18 and C8 cartridges (C18, 102.6% and C8, 93.9%); however, the percentage recoveries of both synthetic testosterone and corticosterone were improved using the C8 cartridge (C18, 68.8 and 65.8% vs. C8, 82.6 and 92.7%, for testosterone and corticosterone, respectively).

### Faecal extract recovery and precision

**Figure 1:**
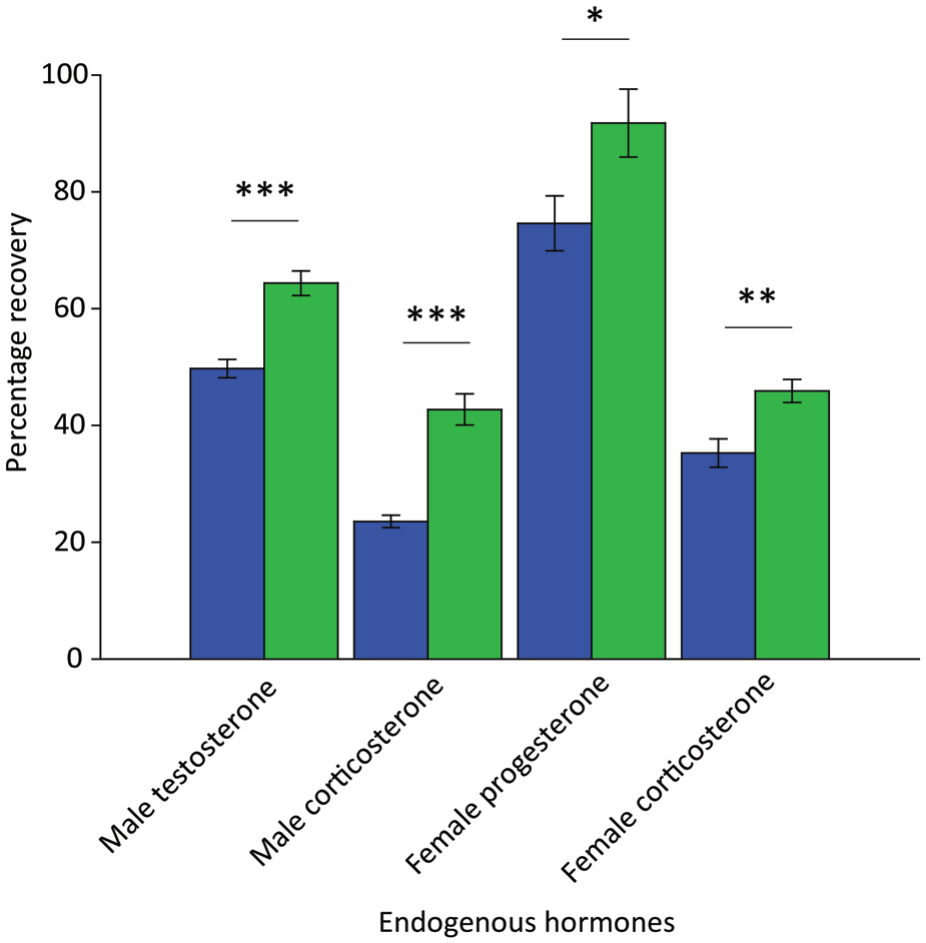
The percentage recovery of black rhinoceros faecal hormone metabolites on two cartridge types (C18, blue bars and C8, green bars). The percentage recovery is calculated as nanograms per gram of faeces measured following the optimal field extraction method, as a percentage of the control laboratory method (*P < 0.05, **P < 0.01 and ***P < 0.001).

Compared with the control laboratory method, the recovery of hormone metabolites from faecal extracts was greater using C8 cartridges for all hormone metabolites tested [for female progesterone, C18, 74% and C8, 89% (*t*_18_ = 2.3, *P* = 0.034); for female corticosterone, C18, 35% and C8, 45% (*t*_18_ = 3.405, *P* = 0.003); for male testosterone, C18, 50% and C8 64% (*t*_18_ = 5.561, *P* < 0.001); and for male corticosterone, C18, 23% and C8, 42% (*t*_18_ = 6.713, *P* < 0.001); Figure 1]. In all cases, the methanol ‘waste’ collected after synthetic hormones or faecal extracts had been loaded on to the SPE cartridges contained negligible concentrations of hormone metabolites, as determined using the respective EIAs, indicating that reduced recovery was not due to failure of the SPE to retain the hormones of interest.

The precision of hormone metabolite concentrations measured following field extraction using C18 cartridges was as follows: progesterone, 15.2% CV; female corticosterone, 16.9% CV; testosterone, 6.55% CV; and male corticosterone, 10.4% CV. Using C8 cartridges, precision was as follows: progesterone, 8.8% CV; testosterone, 11.4% CV; female corticosterone, 9.2% CV; and male corticosterone, 13.9% CV. Based on all of the above findings, C8 cartridges with a loading solvent of 40% were considered the optimal field extraction method and used for the remainder of the study.

### Storage of hormone metabolites

**Figure 2:**
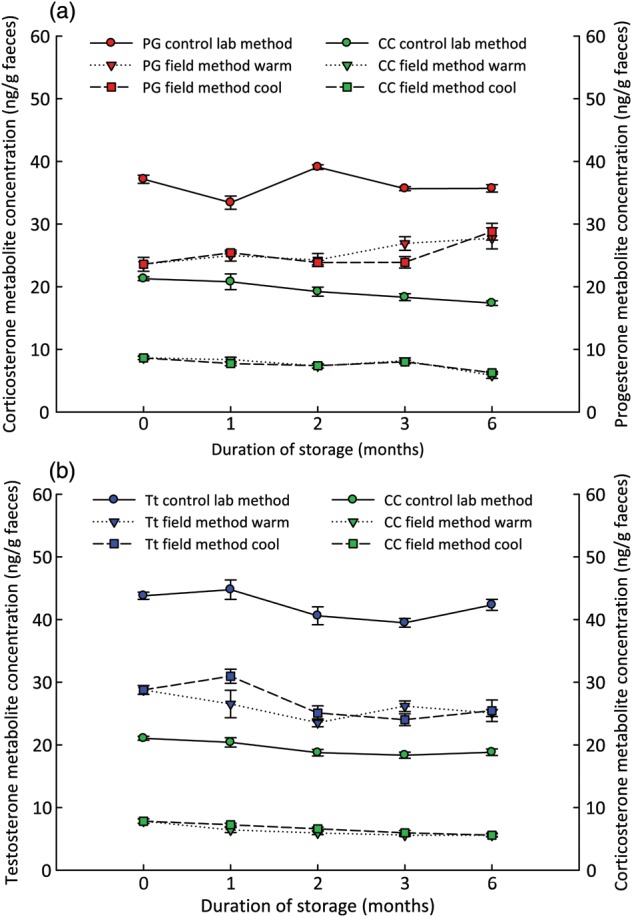
Hormone metabolite concentrations measured from black rhinoceros faeces extracted according to the optimal field extraction method, stored on solid-phase extraction cartridges for 0, 1, 2, 3 and 6 months, and kept in either warm, humid or cool, dry conditions before re-extraction; and compared with the control laboratory method. (**a**) Faecal extracts from a female run on progesterone (PG; red symbols) and corticosterone (CC; green symbols) enzyme immunoassays. (**b**) Faecal extracts from a male run on testosterone (Tt; blue symbols) and corticosterone (CC; green symbols) enzyme immunoassays, expressed as nanograms per gram of faeces.

Progesterone, testosterone and corticosterone metabolites measured in black rhinoceros faecal extracts remained consistent when stored on SPE cartridges for up to 6 months, both when kept in warm, humid and in cool, dry ambient conditions (Figure 2; female progesterone CVs, laboratory 6.43%, warm 11.4% and cool 10.8%; male testosterone CVs, laboratory 7.1%, warm 11.4% and cool 13.3%; female corticosterone CVs, laboratory 10.9%, warm 16.9% and cool 13.2%; male corticosterone CVs, laboratory 7.9%, warm 16.7% and cool 16.0%) for up to 180 days stored on C8 cartridges.

### Comparison of laboratory and field protocols

Overall, faecal progesterone metabolite concentrations were numerically similar when faecal samples were extracted using the two methods (mean ± SD; control laboratory method, 120.86 ± 42.37 ng/g faeces and field method, 115.00 ± 45.69 ng/g faeces). The relative concentrations of both testosterone and corticosterone metabolites obtained using the field extraction method were numerically lower than those obtained by the control laboratory method (testosterone, laboratory, 55.88 ± 11.52 ng/g faeces and field, 26.68 ± 5.72 ng/g faeces; male corticosterone, laboratory, 23.29 ± 7.13 ng/g faeces and field, 9.86 ± 2.83 ng/g faeces; and female corticosterone, laboratory, 41.17 ± 9.00 ng/g faeces and field, 14.34 ± 4.26 ng/g faeces).

**Figure 3:**
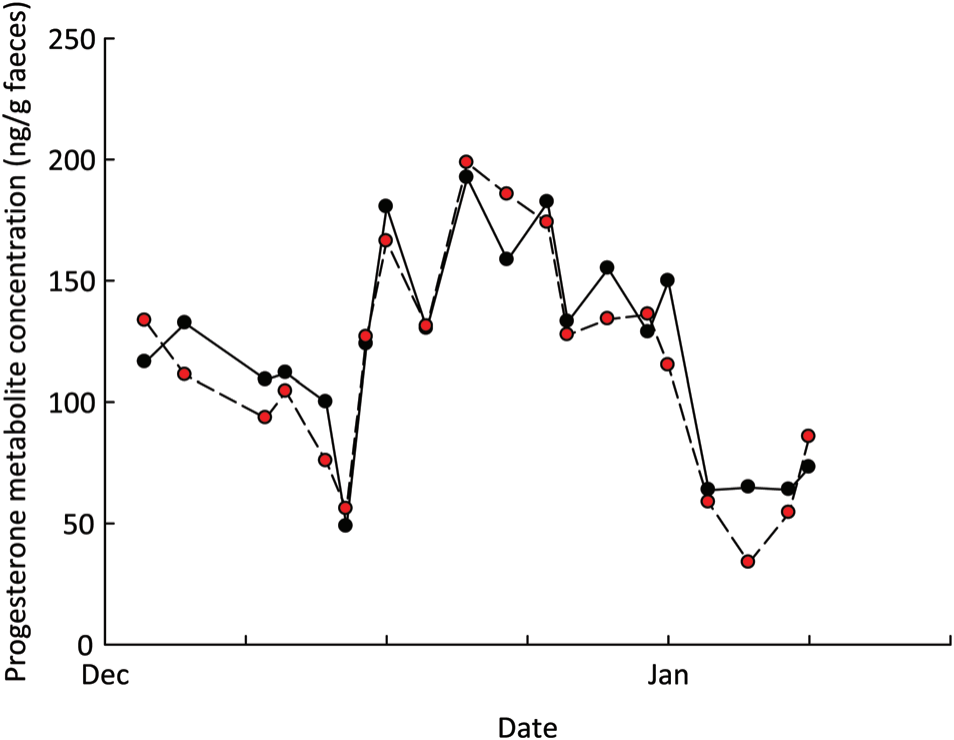
Female black rhinoceros faecal progesterone metabolite concentration (in nanograms per gram of faeces) over one oestrous cycle. Samples were extracted according to either the optimal field extraction method (red circles) or the control laboratory method (black circles).

Although numerically lower in some cases, all hormones extracted using the field method were highly correlated with the control laboratory method (female progesterone, *r* = 0.935, *n* = 20, *P* < 0.001; female corticosterone, *r* = 0.794, *n* = 20, *P* < 0.001; male testosterone, *r* = 0.842, *n* = 20, *P* < 0.001; and male corticosterone, *r* = 0.843, *n* = 20, *P* < 0.001; an example of female progesterone concentrations over one oestrous cycle is illustrated in Figure 3).

## Discussion

The present study demonstrates a practical method by which faecal samples were extracted in field conditions, without the need for extensive equipment or electricity, and loaded onto C8 HyperSep™ SPE cartridges. These cartridges were then stored at ambient temperatures for up to 6 months prior to re-extraction and enzyme immunoassay, without degradation of hormone metabolites. SPE cartridges are compact and lightweight and can easily be transported back to the laboratory following faecal extraction, because they do not contain solvents or raw faecal material. This method could offer a useful alternative for sample preservation when equipment may be limited or electricity supplies may not be available either to freeze samples for long-term storage or to process and dry faecal extracts in the field.

The reduced recovery of hormone metabolites observed using this method was attributed to the SPE cartridges used to store faecal extracts, because the recovery of hormone metabolites did not differ between the faecal extraction method used in the laboratory and the hand-shaking extraction method adopted in the field. Further to previous studies where SPE cartridges have been used as a field extraction technique, we found that although recovery of hormone metabolites overall was reduced compared with the standard laboratory protocol, the recovery of certain hormone metabolites from faecal samples was improved by altering the composition of the solid phase within the cartridge. Using C8 rather than C18 cartridges improved the recovery of faecal glucocorticoid and testosterone metabolites in particular, whereas the recovery of progesterone remained consistently high. Corticosterone and testosterone are more polar compounds than progesterone, which may explain why this difference was observed. The C18 cartridges are better suited to non-polar to moderately polar compounds, whereas the C8 cartridges are slightly less retentive, and thus better suited for a variety of polar and non-polar compounds. We propose that the reduced recovery observed is due to an inability to extract the more polar metabolites fully once stored on the SPE. However, this could not be improved by increasing the volume of solvent used for elution, because multiple elution steps did not improve the recovery of any of the hormone metabolites tested (McArthur, 2011).

This practical method was biochemically validated in male and female black rhinoceros, to measure progesterone, testosterone and glucocorticoid metabolites in faeces by enzyme immunoassay, offering the opportunity to measure both reproductive and adrenal hormones as required. Additionally, a comparison of the standard laboratory and newly developed field protocols demonstrated that although recovery using this field method was consistently lower than using the control laboratory method, particularly for androgen and glucocorticoid metabolites, results were both consistent between replicates and highly correlated with the control laboratory method. This field extraction method revealed qualitatively similar trends in hormone profiles for all three hormone metabolites and delivered a high degree of precision between replicates, all within acceptable limits according to published literature in this field (Munro and Stabenfeldt, 1984; Kurstak, 1985).

The described method of sample extraction and storage could be applied to investigate hormone differences within and between individuals, making it both practical and useful for field researchers investigating reproduction and adrenal activity *in situ*. The use of SPE cartridges for storage may not be limited to storing faecal extracts, but could also be applied to other sample matrices. Furthermore, this versatile method could be used in a wide variety of species and environments and wherever equipment or facilities may be limited.
